# Wnt Signaling Activation in Gingival Epithelial Cells and Macrophages of Experimental Periodontitis

**DOI:** 10.3390/dj11050129

**Published:** 2023-05-09

**Authors:** Ying Chen, Yang Hu

**Affiliations:** Department of Immunology and Infectious Diseases, The Forsyth Institute, 245 First Street, Cambridge, MA 02142, USA

**Keywords:** Wnt signaling, macrophage, periodontitis

## Abstract

**Objective**: Wingless/integrated (Wnt) signaling plays critical roles in maintaining environmental homeostasis and is also involved in the pathogenesis of inflammatory diseases. However, its role in macrophages during periodontitis is not well understood. The present study aims to investigate the interaction between Wnt signaling and macrophages in the context of periodontitis. **Methods**: Experimental periodontitis was induced in C57/BL6 mice using a *Porphyromonas gingivalis* (*P.g*)-associated ligature for 14 days. Immunohistochemistry was performed to study the expression of the pro-inflammatory cytokine tumor necrosis factor (TNF-α), the stabilization of β-catenin, and the macrophage marker F4/80 in the periodontal tissues. The effect of Wnt signaling on TNF-α was examined using Western blot analysis in Raw 264.7 murine macrophages stimulated by Wnt3a-conditioned medium, with or without Wnt3a antibody neutralization, and compared with primary cultured gingival epithelial cells (GECs). The effect of *P.g* lipopolysaccharide (LPS) on Wnt signaling was assessed by analyzing key components of the Wnt signaling pathway, including the activity of low-density lipoprotein receptor-related protein (LRP) 6 and nuclear accumulation of β-catenin in GEC and Raw 264.7 cells. **Results**: Over-expressions of TNF-α and activated β-catenin were presented in the macrophages in the gingiva from mice with *P.g*-associated ligature-induced periodontitis. The expression patterns of TNF-α and activated β-catenin were consistent with the expression of F4/80. In Raw 264.7 cells, activation of the Wnt signaling pathway led to an increase in TNF-α, but this effect was not observed in GEC. Additionally, treatment with LPS induced β-catenin accumulation and LRP6 activation in Raw 264.7 cells, which were blocked by the addition of Dickkopf-1(DKK1). **Conclusions**: Wnt signaling was aberrantly activated in the macrophages in experimental periodontitis. The activation of Wnt signaling in the macrophages may play a pro-inflammatory role in periodontitis. Targeting specific signaling pathways, such as the Wnt pathway, may hold promise for developing novel therapeutic interventions for periodontitis.

## 1. Introduction

Periodontitis is a chronic inflammatory disease that is initiated by reversible infectious gingivitis and develops to irreversible periodontal inflammation. It is a leading cause of jawbone loss and deep tissue damage [1]. Among the various types of cells in the periodontal tissues, gingival epithelial cells (GEC) and macrophages are important cells in maintaining periodontal homeostasis and limiting pathogen invasion [2,3].

The GEC functions as a first line defense against the invasion of exogenous pathogens, protecting the periodontal tissues from biofilm contact. GEC also plays a critical role in triggering and modulating the immune response by expressing several pattern recognition receptors (PRRs) that recognize periodontal pathogens and activate the key inflammatory cytokines to trigger and regulate the immune response of macrophages [4,5]. Gingivitis is the first manifestation of an immune response and contributes to the further development of periodontitis.

Macrophages play a central role in initiating the immune response. A recent study showed that pro-inflammatory macrophages play a pathogenic role in the chronic inflammation associated with periodontitis and is a major resource of pro-inflammatory cytokines. Periodontitis-related inflammation is mainly driven by bacterial communities [6,7], of which *Porphyromonas gingivalis (P.g)* is the most prominent. The virulence factor of *P.g,* lipopolysaccharide (LPS), plays the main pathogenic role in the initiation and enhancing of inflammation [8]. The inflammation begins with the immune response of resident immune cells, including the macrophages to the bacterial biofilm followed by the activation of inflammation cytokines [7]. Among the many pro-inflammatory cytokines that macrophage mediate, tumor necrosis factor α (TNF-α) is one of the key early cytokines induced by periodontal pathogens in destructive periodontitis. TNF-α over-expression in the gingiva has been shown to contribute to the onset of periodontal diseases by augmenting bacterial invasion [9], dysregulating immune responses, inducting other pro-inflammatory mediators and adhesion molecules to facilitate the inflammation response, and promoting the destruction of alveolar bone by stimulating osteoclast formation [9,10,11,12,13].

Canonic Wnt signaling, also referred as the β-catenin-dependent pathway, is highly conserved and essential for development, tissue homeostasis, and disease [14]. This pathway is initiated when Wnt, a family of secreted glycoproteins, binds to the Frizzled (Fz) family of receptors and the co-receptor, low-density lipoprotein receptor-related proteins 5 and 6 (LRP5/6), on the cell surface. This binding leads to the recruitment of the intracellular protein Disheveled (Dvl) to the plasma membrane, disrupting the formation of the destruction complex consisting of Axin, APC, PP2A, and GSK3β [15]. Normally, in the absence of Wnt signaling, the destruction complex is activated, and GSK3β continuously phosphorylates β-catenin at specific sites, including serine 33/37, threonine 41, and serine 45. The phosphorylated β-catenin is then targeted by the β-Trcp E3 ubiquitin ligase complex, leading to its ubiquitination and proteasomal degradation. However, when Wnt signaling is activated, the destruction complex is inhibited, and β-catenin is stabilized and accumulates in the cytoplasm, where it can then translocate to the nucleus and interact with T-cell factor/lymphoid enhancer factor (TCF/LEF) transcription factor to induce the expression of Wnt target genes involved in various cellular processes.

The aberrant activation or dysregulation of the Wnt signaling has been shown to play a key causative mechanistic role in triggering and amplifying pathogenic inflammation [16,17]. In the context of periodontitis, Wnt signaling has been widely recognized as critical for maintaining periodontal tissues and promoting tissue remodeling, particularly in the regeneration of alveolar bone, periodontal ligaments, and cementum [18]. However, the exact role of the canonic Wnt signaling pathway in the inflammation associated with periodontitis in GECs and macrophages remains unclear [19]. In this study, we sought to investigate the role of the canonical Wnt signaling pathway in macrophages and GECs during periodontitis. By examining the interaction between the canonical Wnt signaling pathway and these cell types, we aim to gain insights into the underlying mechanisms of periodontitis and identify potential therapeutic targets.

## 2. Materials and Methods

**Animal:** Wild-type (WT) C57BL/6 mice (Jackson Laboratory) were used for experiments at the age of 8–10 weeks old (body weight 20 g to 28 g) with equal numbers of male and female mice. For the in vitro study, a total 12 mice was used (6 per group). For the in vivo study, a total of 12 mice was used (6 per group). All mice were distributed into groups randomly with different cages/racks in the fully functional animal facility. The Institutional Animal Care and Use Committee approved all the animal experiments (IACUC-20-005). All the animal experiments complied with the ARRIVE guidelines.

**Animal model:** *P.g*-associated ligature-induced experimental periodontitis was induced by ligation with silk (7-0, Fisher Scientific, Waltham, MA, USA) surrounding the maxillary second molars on the left side. The silk was soaked in the *P.g* (*Strain ATCC 33277*) suspension for 30 min before ligation. All the mice were anesthetized by intraperitoneal injection with ketamine/xylazine (87 mg ketamine/kg, 10 mg xylazine/kg). The ligatures were checked every 2 days and replaced if missing. Both groups (Group 1, staining for TNF-α; Group 2, staining for β-catenin; n = 6 animals/group) were sacrificed on day 14, and samples were processed blinded.

**Immunohistochemistry staining:** The maxillae from at least three animals in each treatment groups were harvested and fixed in 4% formaldehyde for 12 h. The samples were then decalcification in 10% EDTA for 2 weeks at 4 °C with agitation. After embedding with paraffin and sectioned along the long axis of the molars, these sections were deparaffinized and rehydrated, followed by antigen retrieval and blocking with 1% BSA (Cell Signaling, Danvers, MA, USA, #9998) and 10% goat serum (Cell Signaling, #5425). Primary antibodies used in the study were rabbit anti-TNFα antibody (Abcam, Cambridge, UK, ab 6671, 1:50), rabbit non-phospho (active) β-catenin at Ser33/37/Thr41 (D13A1) (Cell Signaling, #8814, 1:100), and rabbit anti-mouse F4/80 (MyBiosource, San Diego, CA, USA, MBS8535016, 1:300). A rabbit-specific HRP/DAB kit (Abcam, ab64261) was used to show the antigen localizations. After hematoxylin counterstaining, images of gingival areas mesial and distal to the molars were taken under light microscope.

**Cell preparation and culture:** Mouse gingival epithelial cells were isolated and cultured following a procedure described previously [20]. Briefly, the palatal gingival tissue was minced by scalpel and then put in solutions containing Dispase II (2 mg/mL, Sigma, St. Louis, MO, USA) and collagenase (4 mg/mL, Sigma) for 1.5 h at 37 °C. After centrifugation and rinsing, the cells were separated and cultured in keratinocyte serum-free medium (Gibco, Waltham, MA, USA) for 7 days, after which they were ready to use. To study the direct effect of Wnt on TNF-α, primary cultured gingival epithelial cells and Raw 264.7 cells were stimulated with 0.1 µg/mL *P.g* LPS (Invivogen, tlrl-pglps) for 2 h before being pre-treated with control IgG (R&D system, Minneapolis, MN, USA, 6-001-F, 5 µg/mL) or anti-Wnt3a antibody (Santa Cruz Biotechnology, Dallas, TX, USA, AB_2215415, 5 µg/mL) overnight, then cultured in a control medium or 50% Wnt3a-conditioned medium (ATCC, CRL-2647) for 48 h. To study the effect of *P.g* LPS on the Wnt pathway, gingival epithelial cells and Raw 264.7 murine macrophages were treated with 1 μg/mL *P.g* LPS and were harvested at 0, 1, 3, 6, 12, and 24 h for the Western blot analyses. For the immunohistochemistry analysis, GEC and Raw 264.7 murine macrophages were pretreated with DKK-1 (R&D system, 5897-DK-010/CF, 500 ng/mL) for 24 h, followed by 1 μg/mL *P.g* LPS for 24 h.

### 2.1. Non-Junctional Glycoprotein Isolation

Non-junctional β-catenin was isolated following a protocol previously described by Fagotto et al. [21]. Briefly, after treatment, Raw 264.7 cells and GECs were washed and harvested in a non-ionic detergent buffer, and equal amounts of total protein (50 μg) were incubated with 160 μL concanavalin A (ConA)–magnetic beads (500 μg) for 1 h in an activation buffer (Cell Signaling, Cat. 93569). The Con A beads were then washed 2 times to remove the non-functional cadherin binding protein (junctional β-catenin). The equivalent ConA unbound fraction proteins were subjected to standard SDS-PAGE and immunoblot analysis for Wnt/β-catenin signaling.

### 2.2. Nuclear Fraction Preparation

Raw 264.7 cells and GEC cells were collected, washed, and suspended in a cytoplasmic extract buffer and incubated on ice for 40 min, and then the suspension was centrifuged at 12,000 rpm. The resulting supernatant containing the cytoplasmic extract was carefully collected and stored. The nuclear pellet was washed to remove any residual cytoplasmic material and then resuspended in a nuclear extraction buffer. This suspension was incubated on ice for 15 min and then centrifuged at 14,000 rpm. The resulting supernatant was collected, and the protein concentration of the nuclear extract was determined by a BCA protein assay kit (Thermo Fisher, Cat. 23225) according to the manufacturer’s instructions. The nuclear extracts were then stored at −80 °C until use.

**Immunoblot analysis:** The procedure was followed as described previously [22]. Briefly, nuclear cell lysates or non-junctional cell lysates (20 µg) were used for the analysis of non-phospho β-catenin, and the total cell lysates were used for TNF-α and Phospho LRP6. The expressions were normalized by β-actin levels and analyzed by ImageJ software (Version 1.54d). Specifically, the integrated density of each band of interest was determined, and it was divided by the integrated density of the corresponding β-actin band using ImageJ software. Primary antibodies included rabbit anti-non-phospho β-catenin (Cell Signaling, #8814, 1:500), rabbit anti-TNFα antibody (Abcam, ab 6671, 1:000), rabbit anti-phospho LRP6 (Cell Signaling, #2568, 1:500), and rabbit anti-β-actin (Abcam, ab8227, 1:2000).

**Statistical analysis:** All the results were expressed as means ± SD. Unpaired Student’s *t* tests were used for comparisons of any two groups of data sets in the statistical analysis. Probability values less than 0.05 were considered statistically significant. All statistical analysis was performed by GraphPad Prism.

## 3. Results

### 3.1. The Pro-Inflammatory Cytokine TNF-α Was Elevated in the Periodontal Tissues of Experimental Periodontitis

TNF-α is known to be destructive and is implicated in bone loss during the disease, and there is a strong positive correlation between the over-expression of TNF-α in periodontal tissues and tissue destruction.

**Figure 1:**
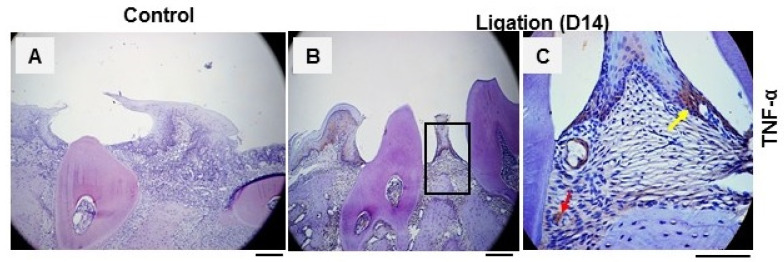
**Elevations of pro-inflammatory cytokine TNF-α in the gingival tissue during periodontitis.** Representative immunohistochemistry analysis of TNF-α expression in periodontal tissues from mice (n = 3) 14 days after ligature induction. (**A**) Non-ligation side periodontal tissues. (**B**) Ligature-induced periodontitis (LIP). (**C**) Enlarged view of the box in (**B**) shows the periodontal tissues. Arrows (red and yellow) indicate the positive staining cells in the gingival tissue. Scale bar: 50 μm.

To better understand the role of TNF-α in periodontitis, we examined its expression in a *P.g*-associated ligature-induced periodontitis model. As depicted in Figure 1, positive staining of TNF-α in the periodontal tissue was discovered in the gingiva of the periodontal region (Figure 1B,C) of mice with periodontitis 14 days after ligation compared to non-ligation control tissue (Figure 1A). These findings indicate that TNF-α in the gingiva may play a crucial role in the pathogenesis by mediating tissue destruction and bone loss; a further mechanism study is therefore needed to determine the cause underlying the inflammation in this disease.

### 3.2. The Canonical Wnt Signal Was Activated in the Gingiva of Experimental Periodontitis

The stabilization and nuclear translocation of β-catenin represents the activation of Wnt signaling, which activates the nuclear transcriptional factor to promote Wnt target gene transcription. TNF-α can activate the Wnt/β-catenin pathway, while the activation of Wnt/β-catenin signaling augments TNF-α and induces NF-kB signaling and pro-inflammatory cytokine expression. Therefore, we examined the key Wnt signaling effector, non-phosphorylated β-catenin (np β-catenin), indicating the activation of the canonical Wnt pathway, in the periodontal tissues during periodontitis.

**Figure 2:**
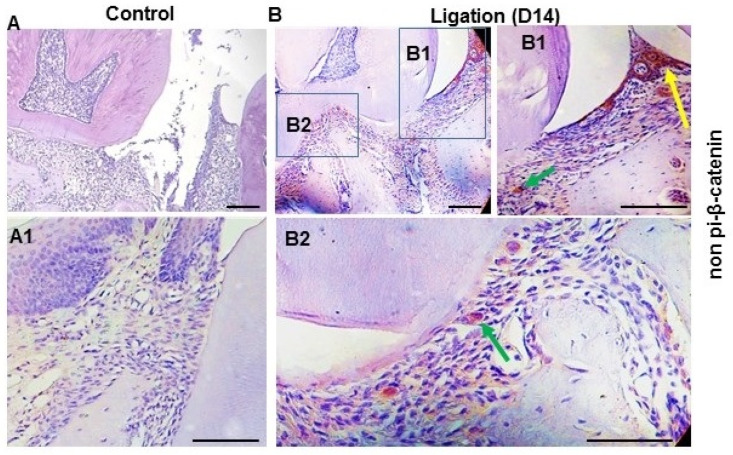
A positive aberrant Wnt signal was activated in the periodontal tissues in experimental periodontitis during periodontitis. Representative immunohistochemical analysis of the non-phosphorylated β-catenin, indicating Wnt signaling activation, in the periodontal section from the mice (n = 3) 14 days after ligation (**B**) and without ligation (**A**), respectively. (**A1**) is one of the enlarged views of without ligation group. (**B1**) is the enlarged view of the box (**B1**) in (**B**), and (**B2**) is the enlarged view of the box (**B2**) in (**B**) to show the positive staining, likely GECs (yellow arrows) or immune cells (green arrows), respectively. Scale bar: 50 μm.

As shown in Figure 2B, the expression of activated β-catenin (non-phosphorylated β-catenin), indicating the activation of Wnt signaling, was high in the gingiva of the mice 14 days after ligation compared with non-ligation control tissue (Figure 2A), and the expression pattern in the gingival was consistent with the expression of the key pro-inflammatory cytokines of TNF-α (Figure 1B). These results together suggested that canonical Wnt signaling was activated and likely involved in the inflammatory process during periodontitis.

These data suggested that the canonical Wnt pathway is likely activated in the gingiva during periodontitis and is consistent with the expression of the key pro-inflammatory cytokines of TNF-α. These results together suggested that canonical Wnt signaling is activated in gingiva, and this activation is involved in the inflammatory process during periodontitis.

### 3.3. Activation of Canonical Wnt Signaling Induced TNF-α Expression in the Macrophages but Not in GEC In Vitro

**Figure 3:**
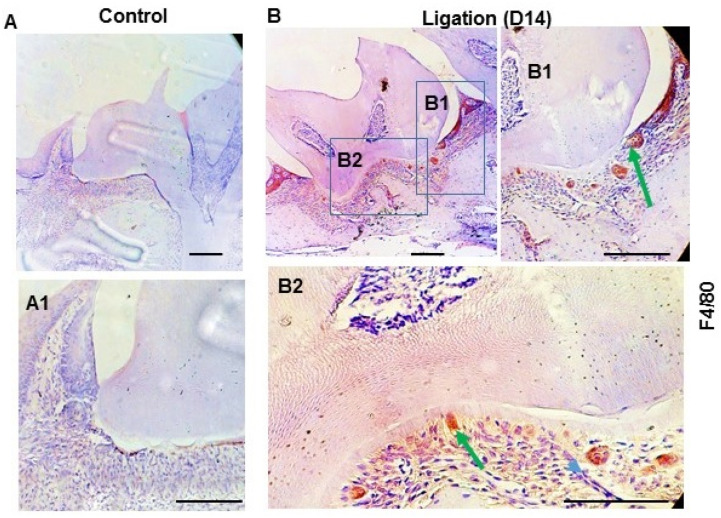
**The expression of macrophage-specific markers in the periodontal tissues during periodontitis. Representative** immunohistochemical staining of the F4/80 in the periodontal section from the mice (n = 3) 14 days after ligation (**B**) and without ligation (**A**), respectively. (**A1**) is one of the enlarged views of without ligation group. (**B1**,**B2**) are the enlarged views of boxes in (**B**). Arrows (green) indicate the positive staining of F4/80. Arrow blue, nuclear staining. Scale bar: 50 μm.

Given the important role that the Wnt signaling pathway plays in recruiting macrophages, we sought to determine whether the pathway is present in macrophages in the gingiva during periodontitis. To this end, we examined the expression of F4/80, a macrophage-specific marker. Our results, shown in Figure 3, revealed a similarity in the expression patterns of F4/80 (Figure 3B), activated β-catenin (Figure 2B), and TNF-α (Figure 1B) in the gingiva during periodontitis. Accordingly, it is reasonable to hypothesize that the consistent expression of activated β-catenin and TNF-α is associated with macrophages, and that the activation of the Wnt pathway is linked to the recruitment and activation of macrophages in the gingiva during periodontitis.

**Figure 4:**
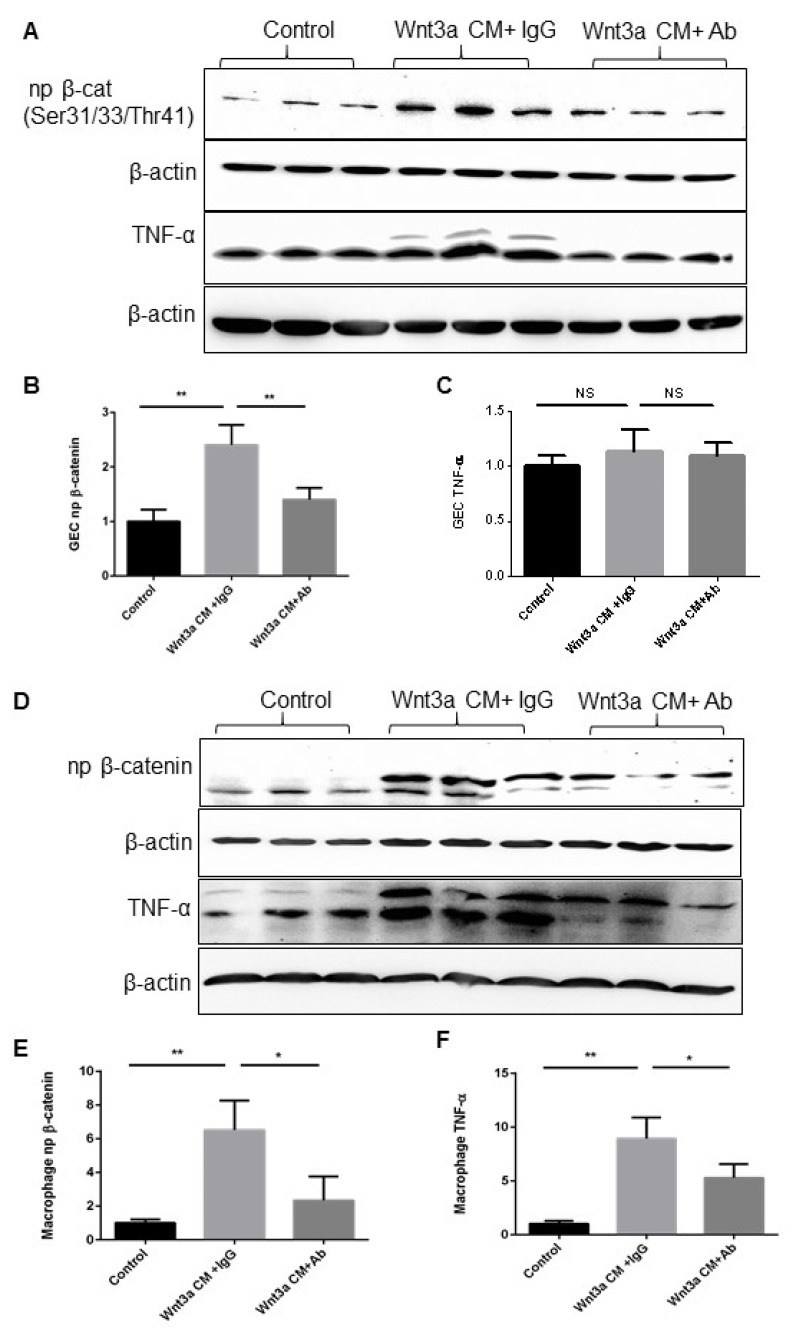
**Activation of Wnt signaling induced the secretion of TNF-α in the GEC and macrophages.** After serum starvation, the primary cultured gingival epithelial cells (**A**) and Raw 264.7 cells (**D**) were pre-treated with control IgG or anti-Wnt3a antibody at a concentration of 5 μg/mL overnight, and then they were cultured in a control medium or 50% Wnt3a-conditioned medium (CM) for 48 h. The non-phosphorylated β-catenin at Ser33/37/Thr41 (**B**,**E**) and the TNF-α (**C**,**F**) were measured by Western blot analysis in equal amounts of non-junctional protein and the total cell lysis in GEC and Raw 264.7 cells, respectively. Each lane represents one individual treatment (mean ± SD; all groups n = 4, * *p* < 0.05, ** *p* < 0.01 as indicated; NS, no significant difference).

To assess if the activation of the Wnt signaling in macrophages plays a pathogenic role in inflammation, we examined if activation of the canonical pathway alone could increase the expression of TNF-α in comparison to primary cultured gingival epithelial cells (GEC). We exposed GECs and Raw 264.7 cells to 50% Wnt3a-conditioned medium (CM), which induced mainly the canonical Wnt signaling pathway, resulting in the accumulation of β-catenin in the nucleus (Figure 4A,C). As shown in Figure 4B,E, exposure to Wnt3a CM for 48 h resulted in the stabilization of non-phosphorylated β-catenin in the nucleus, with a 2.4-fold in GEC and 6.5-fold increase in Raw 264.7 cells, confirming activation of the canonical Wnt pathway.

Next, upon confirmation of Wnt signaling activation, its effects on the expression of TNF-α were subsequently examined and compared, as depicted in Figure 4A,C; interestingly, the levels of TNF-α were not significantly increased in the GEC (Figure 4C) but were significantly increased in the Raw 264.7 cells 8.9 fold (Figure 4F). These data clearly showed a direct association between Wnt signaling activation and the production of TNF-α, and especially in the macrophages but not in the GEC.

To further test the hypothesis that the increase of TNF-α resulted from Wnt signaling activation, we examined whether Wnt-directed TNF-α secretion would be abolished or decreased when Wnt activation was inhibited in these cells. As shown in the representative Western blot in Figure 4A,D, when we neutralized Wnt3a using an anti-Wnt3a-specific antibody, Wnt activation was silenced, as indicated by the decreased accumulations of non-phosphorylated β-catenin in the nucleus from 2.4-fold to 1.4-fold of the control in GEC and from 6.5-fold to 2.3-fold of the control in Raw 264.7 cells. However, unlike the significant alterations of β-catenin in both GEC and Raw 264.7 cells, the change in TNF-α was not significant in GEC (Figure 4C) but significantly decreased in Raw 264.7 cells (Figure 4F). These data suggest that activation of the canonical Wnt signaling has a significant direct effect on the induction of TNF-α in macrophages, but not in GEC.

Overall, these findings support the hypothesis that activation of the Wnt pathway is likely associated with the recruitment and activation of macrophages in the gingiva during periodontitis, which may contribute to the pathogenesis of inflammation in periodontal diseases.

### 3.4. P.g LPS Activated Wnt Signaling in the GEC and Macrophages In Vitro

The principal component from *P.g* is lipopolysaccharide (LPS), which is important virulence factor in the mechanisms of periodontitis and can induce TNF-α secretion. Based on our previous findings, we hypothesized that LPS may also induce Wnt signaling activation in macrophages during periodontitis. To test this hypothesis, we examined the effects of LPS on key components of the Wnt pathway.

**Figure 5:**
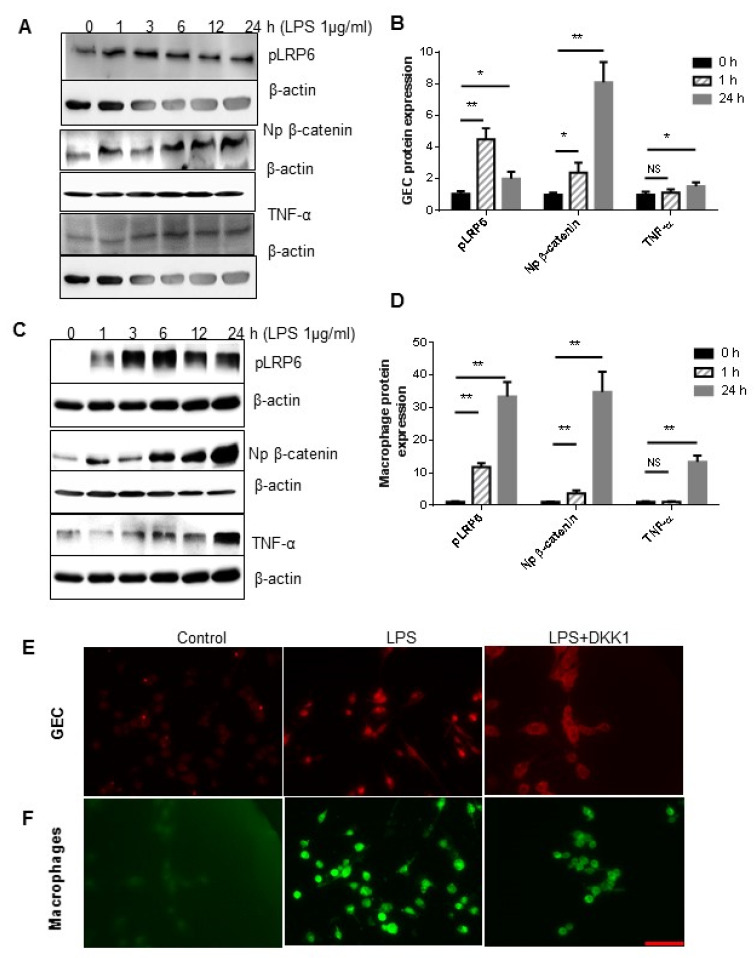
***P.g* LPS induced the activation of Wnt signaling in the GEC and macrophages.** (**A**,**C**) Representative Western blot analysis of pLRP6, β-catenin, and TNF-α in GEC and Raw 264.7 cells. At 70% confluence, primary cultured GEC (**A**) and Raw 264.7 cells (**C**) were stimulated with 1 μg/mL *P.g* LPS for 0 to 24 h, the cells were harvested at indicated time, and the expression of TNF-α and Wnt activation were measured by Western blot analysis. Phosphorylated LRP6 and TNF-α in the equal amount of total cell lysis, and non-phosphorylated β-catenin in the equal amount of non-junctional cell lysis were analyzed and compared at 0, 1, and 24 h in the GEC (**B**) and macrophages (**D**) (mean ± SD; all groups n = 3, * *p* < 0.05, ** *p* < 0.01; NS, no significant difference). Representative immunohistochemistry analysis of the non-junctional β-catenin in the GEC (**E**) and Raw 264.7 cells (**F**) pretreated with 500 ng/mL DKK-1 for 24 h and followed with 1 μg/mL *P.g* LPS for 24 h. Scale bar: 50 μm.

As shown in Figure 5, exposure of GEC (representative Western blots in Figure 5A) and Raw 264.7 cells (representative Western blots in Figure 5C) to LPS (1 μg/mL) for a time range from 1 h to 24 h resulted in increased phosphorylated LRP6, indicating initial Wnt activation, and increased accumulation of non-phosphorylated β-catenin in the nucleus, a hallmark of the canonical Wnt signaling activation (Figure 5B,D).

To further investigate the effect of LPS-induced Wnt signaling activation on TNF-α production, we used DKK1, a Wnt antagonist via the internalization of the key co-receptor LRP6 on the cell surface, to inhibit Wnt signaling in GEC and macrophages. As shown in Figure 4E,F, pretreatment with 500 ng/mL of DKK1 for 24 h prevented the nuclear accumulation of non-junctional β-catenin in both GEC and Raw 264.7 cells upon stimulation with LPS (1 μg/mL for 2 h), indicating inhibition of canonical Wnt signaling. Interestingly, the levels of TNF-α were not significantly altered in GEC (Figure 5B) but were remarkably increased in Raw 264.7 cells upon LPS stimulation (Figure 5D). Moreover, the increase of phosphorylated LRP6 and non-phosphorylated β-catenin in Raw 264.7 cells was significantly higher than in GEC.

Taken together, these results suggest that LPS functions as a strong activator of the Wnt pathway in both GEC and macrophages. However, Wnt signaling activation in macrophages, but not in GEC, likely plays a major pro-inflammatory role.

## 4. Discussion

Periodontitis is a complex disease caused by the interaction between the oral microbiota and the host immune system, resulting in the destruction of the periodontal tissues and alveolar bone loss. In our present study, we focused on Wnt pathway activation and its relationship with TNF-α, a key pro-inflammatory cytokine in periodontitis. Our findings suggest that Wnt signaling is activated in the gingival tissue during periodontitis induced by *P.g* LPS ligature. We also demonstrated that the Wnt pathway has a direct pro-inflammatory effect on the production of TNF-α in macrophages in the gingival tissue. Furthermore, we found that LPS can activate the Wnt pathway, while inhibition of the Wnt signaling results in the reduction of TNF-α in macrophages.

The present study investigated the role of TNF-α and Wnt signaling in the pathogenesis of destructive periodontitis. The findings of this study are consistent with previous research that has shown TNF-α to be a potent early pro-inflammatory cytokine in destructive periodontitis, and that its activities in the periodontal tissues include degradation of the connective tissues and stimulation of osteoclast formation [23]. Previous research has also suggested a complex interaction between Wnt signaling and TNF-α in various cells. For instance, Jang et al. demonstrated that TNF-α activates the Wnt/β-catenin pathway in human bronchial epithelial cells [24], while Hiyama showed a complex interaction between Wnt signaling and TNF-α in nucleus pulposus cells. In the latter study, the activation of Wnt signaling up-regulated TNF-α expression and caused cell degeneration [25,26]. In the present study, we found that although the expression of TNF-α and the aberrant activated signaling were concurrent in the GEC and macrophages during periodontitis, the regulatory role of Wnt signaling on TNF-α was different in these two cell types. In the GEC, activation or inhibition of Wnt signaling generated insignificant changes in TNF-α expression. In contrast, in the Raw 264.7 macrophage cell line, Wnt signaling activation caused a robust increase in TNF-α, while blocking the Wnt pathway resulted in a dramatic reduction. These disparities suggest that macrophages play a crucial role in stimulating TNF-α secretion during periodontitis, which is consistent with the previous findings that macrophages are a major source of TNF-α [3]. Further studies are needed to elucidate the mechanisms underlying this differential regulatory effect.

The study also found that *P.g* LPS activated Wnt signaling in macrophages, which likely induces M1 polarization based on its strong TNF-α induction effect. Wnt signaling has been shown to play a protective role in the development and maintenance of periodontal tissues and regeneration of the alveolar bone, periodontal ligament, and cementum [27]. Therefore, modulation of macrophage polarization through interfering with Wnt signaling may indicate a novel direction to prevent the inflammation and bone loss in periodontal tissue.

Although the study did not find significant alteration of TNF-α expression upon activation of Wnt signaling, the present study did find interesting disparities in the accumulation of β-catenin and the phosphorylation of LRP6 during a time course, GEC displayed a rapid response to the stimulator, and the role of the Wnt signaling in GEC acting as an inducer or a messenger through the secretion of Wnt ligand or TNF-α for macrophages is unclear. The present study also showed that LPS acted as an activator of the Wnt pathway, likely through interfering with the binding of the Wnt ligand to the receptor or post-translational modification of the receptor. Further research is necessary to explore potential crosstalk between the Wnt and toll-like receptor signaling pathways, which both induce pro-inflammatory cytokines.

The findings of this study contribute to our understanding of the complex interactions between TNF-α and Wnt signaling in periodontitis. Previous research has highlighted the significant role of TNF-α in the pathogenesis of destructive periodontitis, and our study found that activated Wnt signaling plays a complex and cell-type specific role in TNF-α expression, suggesting a modulating effect on inflammation during periodontitis.

It is important to note that this study has some limitations. Firstly, we focused exclusively on the effects of Wnt signaling on TNF-α expression and did not investigate the potential influence of other inflammatory cytokines or signaling pathways. Given the complexity of periodontitis, it is likely other signaling pathways are involved and may cross-talk with Wnt signaling. Secondly, our experimental periodontitis mice model was limited to a 14-day period. While this time frame allowed us to observe significant changes in TNF-α expression, further research is needed to investigate the progression of periodontitis over shorter or longer periods. This would provide a more comprehensive understanding of the disease process. Additionally, we attempted to perform quantitative analysis of the images, but the complex and variable background made it impossible to obtain accurate and reproducible results. Therefore, we presented the data in a binary representation format, indicating either the presence or absence of staining, in order to provide a clear indication of the staining pattern, but it does have its limitations.

In addition, in Figure 4B–F and Figure 5, it appears that there is a difference in the TNF-α effect between the control and the Wnt3a+ Ab group (Figure 4) or between 1 h and 24 h in Figure 5. There may be several factors contributing to this difference. One possibility is that Wnt signaling is not completely blocked in the Wnt3a+Ab group, leading to some residual Wnt activity. Another possibility is that non-Wnt pathways may be activated downstream of the receptor, bypassing the need for Wnt signaling altogether. These potential mechanisms make the interpretation of the results complicated and require further study to explore. Therefore, in our present study, we aim to focus solely on comparing the effect of Wnt activation and inactivation, without delving into the intricacies of the downstream signaling pathways.

Overall, our findings shed new insights into the interplay between inflammation and Wnt signaling in macrophages during periodontitis and highlight the need for further research to fully understand the mechanism involved in this complex disease. Targeting specific signaling pathways, such as the Wnt pathway, may hold promise for developing novel therapeutic interventions for periodontitis. We hope our study will contribute the development of new treatments for this challenging condition.

## References

[1] Howard K.C., Gonzalez O.A., Garneau-Tsodikova S. (2021). Porphyromonas gingivalis: Where do we stand in our battle against this oral pathogen?. RSC Med. Chem..

[2] Maksylewicz A., Bysiek A., Lagosz K.B., Macina J.M., Kantorowicz M., Bereta G., Sochalska M., Gawron K., Chomyszyn-Gajewska M., Potempa J. (2019). BET Bromodomain Inhibitors Suppress Inflammatory Activation of Gingival Fibroblasts and Epithelial Cells From Periodontitis Patients. Front. Immunol..

[3] Fageeh H.I., Fageeh H.N., Patil S. (2021). Monocyte Differentiation into Destructive Macrophages on In Vitro Administration of Gingival Crevicular Fluid from Periodontitis Patients. J. Pers. Med..

[4] Sarah S.M., Tamilselvan S., Kamatchiammal S., Suresh R. (2006). Expression of Toll-like receptors 2 and 4 in gingivitis and chronic periodontitis. Indian J. Dent. Res..

[5] Zhang F., Yang X.M., Jia S.Y. (2020). Characteristics of neutrophil extracellular traps in patients with periodontitis and gingivitis. Braz. Oral. Res..

[6] Shaddox L.M., Goncalves P.F., Vovk A., Allin N., Huang H., Hou W., Aukhil I., Wallet S.M. (2013). LPS-induced inflammatory response after therapy of aggressive periodontitis. J. Dent. Res..

[7] Mahanonda R., Sa-Ard-Iam N., Charatkulangkun O., Promsudthi A., Schifferle R.E., Yongvanichit K., Pichyangkul S. (2004). Monocyte activation by Porphyromonas gingivalis LPS in aggressive periodontitis with the use of whole-blood cultures. J. Dent. Res..

[8] Xu W., Zhou W., Wang H., Liang S. (2020). Roles of Porphyromonas gingivalis and its virulence factors in periodontitis. Adv. Protein. Chem. Struct. Biol..

[9] Hoare A., Soto C., Rojas-Celis V., Bravo D. (2019). Chronic Inflammation as a Link between Periodontitis and Carcinogenesis. Mediat. Inflamm.

[10] Yu Y.Q., Qu L., Qiu L.H., Guo J.J., Ma N., Zhu L. (2016). Mechanism of TNF-alpha in bone defect of chronic apical periodontitis. Shanghai Kou Qiang Yi Xue.

[11] Pathak J.L., Fang Y., Chen Y., Ye Z., Guo X., Yan Y., Zha J., Liang D., Ke X., Yang L. (2021). Downregulation of Macrophage-Specific Act-1 Intensifies Periodontitis and Alveolar Bone Loss Possibly via TNF/NF-kappaB Signaling. Front Cell Dev. Biol..

[12] Kanzaki H., Makihira S., Suzuki M., Ishii T., Movila A., Hirschfeld J., Mawardi H., Lin X., Han X., Taubman M.A. (2016). Soluble RANKL Cleaved from Activated Lymphocytes by TNF-alpha-Converting Enzyme Contributes to Osteoclastogenesis in Periodontitis. J. Immunol..

[13] Beklen A., Ainola M., Hukkanen M., Gurgan C., Sorsa T., Konttinen Y.T. (2007). MMPs, IL-1, and TNF are regulated by IL-17 in periodontitis. J. Dent. Res..

[14] Hayat R., Manzoor M., Hussain A. (2022). Wnt signaling pathway: A comprehensive review. Cell Biol. Int..

[15] Albrecht L.V., Tejeda-Munoz N., De Robertis E.M. (2021). Cell Biology of Canonical Wnt Signaling. Annu. Rev. Cell Dev. Biol..

[16] Zhou Y.Q., Tian X.B., Tian Y.K., Mei W., Liu D.Q., Ye D.W. (2022). Wnt signaling: A prospective therapeutic target for chronic pain. Pharmacol. Ther..

[17] Zou G., Park J.I. (2022). WNT Signaling in Liver Regeneration, Disease, and Cancer. Clin. Mol. Hepatol..

[18] Napimoga M.H., Nametala C., da Silva F.L., Miranda T.S., Bossonaro J.P., Demasi A.P., Duarte P.M. (2014). Involvement of the Wnt-beta-catenin signalling antagonists, sclerostin and dickkopf-related protein 1, in chronic periodontitis. J. Clin. Periodontol..

[19] Chatzopoulos G.S., Koidou V.P., Wolff L.F. (2022). Expression of Wnt signaling agonists and antagonists in periodontitis and healthy subjects, before and after non-surgical periodontal treatment: A systematic review. J. Periodontal. Res..

[20] Chen Y., Jiang Z., Keohane A., Hu Y. (2022). In vitro and in vivo study of the pathogenic role of PPARalpha in experimental periodontitis. J. Appl. Oral. Sci..

[21] Fagotto F., Funayama N., Gluck U., Gumbiner B.M. (1996). Binding to cadherins antagonizes the signaling activity of beta-catenin during axis formation in Xenopus. J. Cell Biol..

[22] Yu P., Hu Y., Liu Z., Kawai T., Taubman M.A., Li W., Han X. (2017). Local Induction of B Cell Interleukin-10 Competency Alleviates Inflammation and Bone Loss in Ligature-Induced Experimental Periodontitis in Mice. Infect Immun..

[23] Chen L., Bao J., Yang Y., Wang Z., Xia M., Tan J., Zhou L., Wu Y., Sun W. (2020). Autophagy was involved in tumor necrosis factor-alpha-inhibited osteogenic differentiation of murine calvarial osteoblasts through Wnt/beta-catenin pathway. Tissue Cell.

[24] Jang J., Jung Y., Chae S., Chung S.I., Kim S.M., Yoon Y. (2017). WNT/beta-catenin pathway modulates the TNF-alpha-induced inflammatory response in bronchial epithelial cells. Biochem. Biophys Res. Commun..

[25] Hiyama A., Yokoyama K., Nukaga T., Sakai D., Mochida J. (2015). Response to tumor necrosis factor-alpha mediated inflammation involving activation of prostaglandin E2 and Wnt signaling in nucleus pulposus cells. J. Orthop. Res..

[26] Hiyama A., Yokoyama K., Nukaga T., Sakai D., Mochida J. (2013). A complex interaction between Wnt signaling and TNF-alpha in nucleus pulposus cells. Arthritis Res. Ther..

[27] Kovacs B., Vajda E., Nagy E.E. (2019). Regulatory Effects and Interactions of the Wnt and OPG-RANKL-RANK Signaling at the Bone-Cartilage Interface in Osteoarthritis. Int. J. Mol. Sci..

